# Supplementary material for: Calretinin Functions in Malignant Mesothelioma Cells Cannot Be Replaced by the Closely Related Ca2+-Binding Proteins Calbindin-D28k and Parvalbumin
Source: Int J Mol Sci. 2018 Dec 12;19(12):4015. doi: 10.3390/ijms19124015 (PMC6321210; doi:10.3390/ijms19124015)
Supplement: Supplementary file 1 [file ijms-19-04015-s001.pdf]

International Journal of *Molecular Sciences*

MDPI

Supplemental Material

Supplemental Methods: Migration and invasion assays

Migration and invasion of SPC111 wt and CB-expressing cells was assessed *in vitro* as described before [13]. Briefly, cells were grown to confluence in 96-well ImageLock plates (Essen Bioscience) pre-coated with a thin layer of 0.1 mg/mL Matrigel Basement Membrane Matrix (Corning, Cat. No.354234). A scratch was created using the 96 well-plate woundmaker tool (Essen Bioscience). For the invasion assay, 50 µL of Matrigel (1 mg/mL) was added to each well. Migration and invasion were monitored using the Incucyte™ system (Essen Bioscience).

Supplemental Figures

A

Clones	ng CaBP/mg of protein extract	Molarity	µM	Ca^{2+} binding
**CR1**	101.6	6.3×10^{-4}	630.8	3154
**CR2**	31.7	1.9×10^{-4}	197.0	985
**CR3**	19.2	1.2×10^{-4}	119.2	596
**CR5**	74.9	4.6×10^{-4}	464.8	2324
**CB2**	32	2.1×10^{-4}	208.0	832
**CB4**	32	2.1×10^{-4}	208.6	835
**PV1**	0.2	3.4×10^{-6}	3.4	7
**PV2**	0.3	5.3×10^{-6}	5.3	11
**PV3**	0.5	7.8×10^{-6}	7.8	16

B

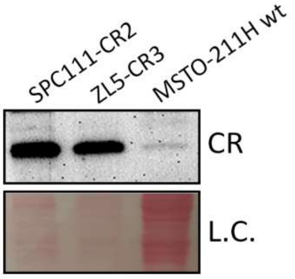

Figure S1. Estimation of the total Ca^{2+}-binding capacity in ZL5 cells and Western Blot analysis of CR levels in CR-expressing clones compared to endogenous CR levels in MSTO-211H wt cells. A. Estimated intracellular concentrations in ZL5 CaBP-overexpressing clones. B. Western Blot analysis of SPC111-CR2 and ZL5-CR3 clones and MSTO-211H wt cells. 5 µg of protein extract was loaded in the case of SPC111-CR2 and ZL5-CR3, and 20 µg in the case of MSTO-211H wt cells. Red Ponceau Red staining intensity is shown in the figure (L.C.).

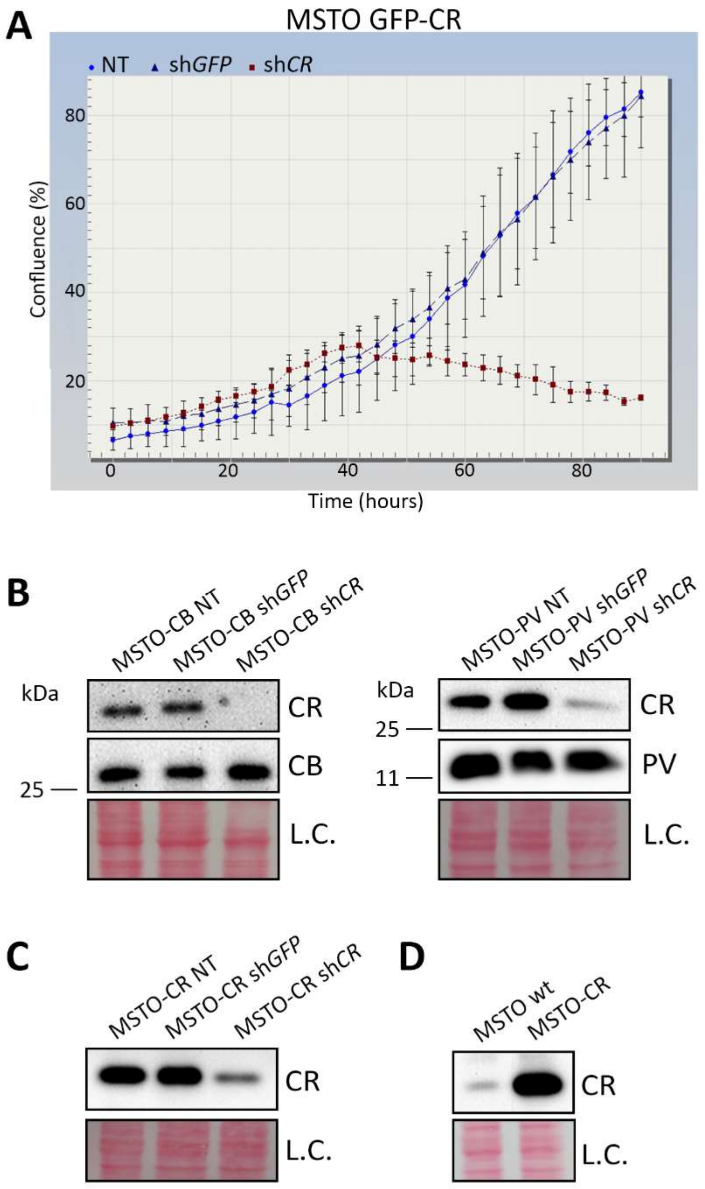

Figure S2. Growth curves of MSTO GFP-CR cells and Western Blot analysis of MSTO-CR, -CB and -PV cells treated with sh*GFP* and sh*CR.* A. Comparison of growth curves of MSTO GFP-CR cells non-treated (NT), treated with an sh*GFP* or an sh*CR*. While no differences are observed in the growth curves between NT (light blue) and sh*GFP*-treated cells (dark blue), cells treated with an sh*CR* (red) show a clear decrease at the proliferation rate. B. Western Blot analysis of protein extracts of MSTO-CB and -PV cells NT, treated with an sh*GFP* and an sh*CR* for 96 h. No changes of CB or PV protein levels are detected after any of the treatments. The positions of the protein size markers are shown in the figure. Ponceau Red staining intensity was used as loading control (L.C.). C. Western Blot analysis of protein extracts of MSTO-CR cells non-treated (NT), treated with an sh*GFP* and an sh*CR* for 96 h. A decrease (>50%) of CR protein levels is observed in MSTO-CR cells treated with the sh*CR*. D. Western Blot analysis of MSTO wt and CR-overexpressing MSTO-CR cells.

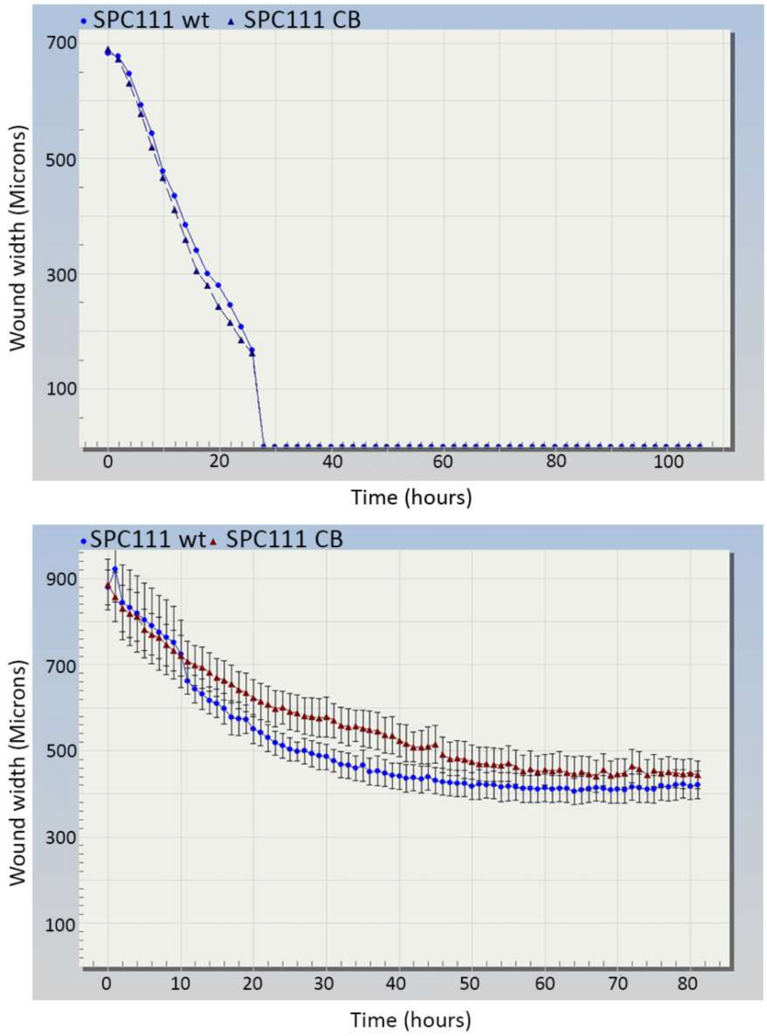

Figure S3. Comparison of wound closure kinetics between SPC111 wt and CB-overexpressing cells ("bulk" population) monitored with the IncuCyte™ imaging system (migration, upper graph; invasion, lower graph). No differences were observed in the closure kinetics between wt and CB-expressing cells (n = 3 independent experiments).
